# Transcriptome Analysis Reveals the Gene Expression Changes in the Silkworm (*Bombyx mori*) in Response to Hydrogen Sulfide Exposure

**DOI:** 10.3390/insects12121110

**Published:** 2021-12-13

**Authors:** Rui Zhang, Yu-Yao Cao, Juan Du, Kiran Thakur, Shun-Ming Tang, Fei Hu, Zhao-Jun Wei

**Affiliations:** 1 School of Food and Biological Engineering, Hefei University of Technology, Hefei 230009, China; 2020111323@mail.hfut.edu.cn (R.Z.); cyy199574@mail.hfut.edu.cn (Y.-Y.C.); kumarikiran@hfut.edu.cn (K.T.); 2 School of Biological Science and Engineering, North Minzu University, Yinchuan 750021, China; 20217528@stu.nun.edu.cn; 3 Jiangsu Key Laboratory of Sericultural Biology and Biotechnology, School of Biotechnology, Jiangsu University of Science and Technology, Zhenjiang 212003, China; tangsm1971@just.edu.cn; 4 Key Laboratory of Silkworm and Mulberry Genetic Improvement, Ministry of Agriculture, Sericultural Research Institute, Chinese Academy of Agricultural Sciences, Zhenjiang 212018, China

**Keywords:** silkworm, hydrogen sulfide, transcriptome, differentially expressed genes, expression pattern

## Abstract

**Simple Summary:**

The fat body is one of the most important tissues in the body of insects due to its number of functions. Nowadays the new physiological function of H_2_S has gained attention as a novel signaling molecule. H_2_S performs crucial regulatory functions involving growth, the cardiovascular system, oxidative stress, and inflammation in many organisms. In this study, RNA-seq technology was used to investigate the fat body of the silkworm at the transcriptional level after H_2_S exposure during the 5th larvae stage. A total of 1200 (DEGs) was identified after 7.5 µM H_2_S treatment, of which 977 DEGs were up-regulated and 223 DEGs were down-regulated. DEGs were mainly involved in the transport pathway, cellular community, carbohydrate metabolism, and immune-associated signal transduction. Present research provides new insights on the gene expression changes in the fat body of silkworms after H_2_S exposure.

**Abstract:**

Hydrogen sulfide (H_2_S) has been recognized for its beneficial influence on physiological alterations. The development (body weight) and economic characteristics (cocoon weight, cocoon shell ratio, and cocoon shell weight) of silkworms were increased after continuous 7.5 µM H_2_S treatment. In the present study, gene expression changes in the fat body of silkworms at the 5th instar larvae in response to the H_2_S were investigated through comparative transcriptome analysis. Moreover, the expression pattern of significant differentially expressed genes (DEGs) at the 5th instar larvae was confirmed by quantitative real-time PCR (qRT-PCR) after H_2_S exposure. A total of 1200 (DEGs) was identified, of which 977 DEGs were up-regulated and 223 DEGs were down-regulated. Most of the DEGs were involved in the transport pathway, cellular community, carbohydrate metabolism, and immune-associated signal transduction. The up regulated genes under H_2_S exposure were involved in endocytosis, glycolysis/gluconeogenesis, the citrate cycle (TCA cycle), and the synthesis of fibroin, while genes related to inflammation were down-regulated, indicating that H_2_S could promote energy metabolism, the transport pathway, silk synthesis, and inhibit inflammation in the silkworm. In addition, the expression levels of these genes were increased or decreased in a time-dependent manner during the 5th instar larvae. These results provided insight into the effects of H_2_S on silkworms at the transcriptional level and a substantial foundation for understanding H_2_S function.

## 1. Introduction

The silkworm, *Bombyx mori*, is a pivotal model of Lepidoptera, a holometabolous insect, with a considerable economic importance in the world. The silkworm has been considered a model animal because of its appropriate life cycle, cultivation, and fast reproduction [1,2]. The fat body for insects acts as the dynamic tissue and the metabolic organ, mainly involved in synthesis, nutrient storage, and energy metabolism [3], and it has been widely studied in various studies of silkworm to reflect various biological, physiological, and biochemical processes. The over expression of BmFoxO in the fat body could suppress protein translation in transgenic silkworms [4].

The toxic effects of hydrogen sulfide (H_2_S) as an environmental pollutant have been studied in the past years. However, nowadays, the new physiological function of H_2_S has gained attention as a novel signaling molecule. H_2_S performs crucial regulatory functions involving growth, the cardiovascular system, oxidative stress, and inflammation in many organisms [5,6]. H_2_S has been accepted as the third transmission gas followed by nitric oxide (NO) and carbon monoxide (CO) in past years with applications to various insects. Miller and Roth (2007) found that treatment with high concentrations of H_2_S are toxic to *Caenorhabditis elegans*; However, exposure to low concentrations of H_2_S increased the thermotolerance and lifespan via increasing the activity of SIR-2.1 [7]. Budde and Roth (2010) found that hif-1 is required when *C. elegans* were exposed to H_2_S; in turn, H_2_S could increase both HIF-1 protein concentration and nuclear localization [8]. H_2_S is an endogenous regulator of oxidative damage, metabolism, and aging in *C. elegans* [9]. Wei and Kenyon (2016) found that removing germ cells in *C. elegans* triggers a level of hydrogen sulfide in nonreproductive somatic tissues. Hydrogen sulfide displayed the protective responses that slow aging [10]. H_2_S treatment significantly increased the survival and lifespan of *Drosophila melanogaster* under arid and food-free conditions [11,12].

In our previous research, it was found that the development (body weight) and economic characteristics (cocoon weight, cocoon shell ratio, and cocoon shell weight) of silkworm (*Bombyx mori*) were increased after continuous treatment with 7.5 μM of H_2_S. After exposure to H_2_S, the level of metabolites related to inflammation ((6Z,9Z,12Z)-octadecatrienoic acid, hexadecanoic acid, choline phosphate, and malic acid, etc.) in the hemolymph of silkworms were significantly increased compared to control group [13]. However, when the concentrations of H_2_S were higher than 7.5 μM, H_2_S displayed toxic effects to the silkworm, including a decrease in body weight, cocoon weight, cocoon shell weight, etc. RNA-seq technology is widely used in the field of silkworms to investigate physiological and biochemical changes at the transcriptional level under diverse conditions [14]. However, there is no effective report for the effects of H_2_S on silkworms at the transcriptional level.

The present study aimed to investigate the expression level of genes in the fat body of silkworms after exposure to H_2_S. The results based on the transcriptomic and bioinformatic analysis showed significantly different responses to H_2_S and provided useful insights into the role of H_2_S on silkworms.

## 2. Materials and Methods

### 2.1. Silkworm Rearing and H2S Exposure

P50 strains of silkworm were obtained from the Sericulture Research Institute of the Chinese Academy of Agricultural Sciences, Zhenjiang, China. According to the previous study [13], from the 4th instar larvae to the 5th instar larvae, silkworms were divided into two groups (H_2_S-treated and control) and reared in desiccators using fumigating treatment at 25 ± 1 °C and 12L:12D photoperiod conditions. Silkworms in the H_2_S-treated group were treated by NaHS (Shandong West Asia Chemical Company, Jinan, China), a donor of H_2_S. The experimental concentration of H_2_S was set to 7.5 μM according to the report of Cao et al. (2020) [13]. There were triplicates in each group with 30 silkworms per replicate and the silkworms were fed abundant fresh mulberry leaves twice a day.

### 2.2. RNA Extraction

The total RNA of the silkworm fat body was extracted on ice from the 1st day (5L1D) to the 5th day (5L5D) at the 5th instar larvae using Trizol reagent (Invitrogen, CA, USA). The concentration and purity of total RNA were analyzed by NanoDrop ND-1000 (NanoDrop, Wilmington, DE, USA) and the ratios of A260/A280 and 260/230 were used to measure the quality of total RNA. 

### 2.3. Sequencing, Data Processing, and Assembly

The total RNA of 5L3D was analyzed in the H_2_S-treated and control groups by RNA-Sequencing. Total RNA (10 µg) of each sample was subjected to Poly(A) mRNA isolation with poly-T oligo-attached magnetic beads (Invitrogen, CA, USA). The poly(A) RNA was fragmented into small pieces using a Magnesium RNA Fragmentation Module (New England Biolabs (Beijing) LTD., cat. e6150, Beijing, China) under 94 °C for 5–7 min. The cleaved RNA fragments were then reverse-transcribed to create the cDNA by SuperScript^TM^ II Reverse Transcriptase (Invitrogen, CA, USA), which were next used to synthesize U-labeled second-stranded DNAs. An A-base was then added to the blunt ends of each strand, preparing them for ligation to the indexed adapters. Each adapter contained a T-base overhang for ligating the adapter to the A-tailed fragmented DNA. Single- or dual-index adapters were ligated to the fragments, and size selection was performed with AMPureXP beads. After the heat-labile UDG enzyme (New England Biolabs (Beijing) LTD., cat.m0280, Beijing, China) treatment of the U-labeled second-stranded DNAs, the ligated products were amplified with PCR by the following conditions: initial denaturation at 95 °C for 3 min; 8 cycles of denaturation at 98 °C for 15 s, annealing at 60 °C for 15 s, and extension at 72 °C for 30 s; and final extension at 72 °C for 5 min. The average insert size for the final cDNA library was 300 ± 50 bp. Lastly, we performed the 2 × 150 bp paired-end sequencing (PE150) on an Illumina Novaseq^TM^ 6000 (LC-Bio Technology CO., Ltd., Hangzhou, China) following the vendor’s recommended protocol. 

The transcriptome was sequenced on basis of the Illumina paired-end RNA-seq approach. The average insert size for the paired-end libraries was 300 bp (±50 bp). The low-quality reads with sequencing adaptors, sequencing primer, and nucleotide (q quality score (*p* value corrected by BH algorithm, also called FDR value, *q* value, *p* adj value) <20) were removed using Cutadapt software (https://cutadapt.readthedocs.io/en/stable/, accessed on 1 August 2021) [15] before assembly. We used HISAT2 software (https://daehwankimlab.github.io/hisat2/,version:hisat2-2.0.4, accessed on 1 August 2021) [16] to map reads to the genome. Three important parameters, such as Q20 (the percentage of bases with mass value ≥20), Q30 (mass value ≥30), and GC content were verified to evaluate all the reads using the FastQC online tool [17].

The *Bombyx mori* reference genome [18] was used for the alignment of the sample reads using the HISAT package [19]. StringTie (http://ccb.jhu.edu/software/stringtie/, accessed on 1 August 2021) was used for the assembly of the mapped reads of each sample and a comprehensive transcriptome was constructed combining all transcriptomes using Perl scripts [20]. The expression level of mRNAs was used to calculate the fragments per kilobase of exon model per million mapped reads (FPKM) by StringTie (http://www.bioconductor.org/packages/release/bioc/html/ballgown.html, accessed on 1 August 2021) [20]. The FPKM could evaluate the abundance of genes. The DEGs were selected with log_2_ (fold change) >1 or log_2_ (fold change) <−1 at *p*-value < 0.05, and *q*-value > *p*-value by R package-edgeR (https://bioconductor.org/packages/release/bioc/html/edgeR.html, accessed on 1 August 2021) [21].

### 2.4. Functional Annotation and Enrichment Analysis of DEGs

The reference sequences for all the silkworm genes were obtained from the NCBI database and BLASTX was used for annotation. All the sequences were aligned against Gene Ontology (GO) and Kyoto Encyclopedia of Genes and Genomes (KEGG) databases using DIAMOND (0.7.12) [22]. GO function or KEGG pathway significant enrichment analysis first mapped all the DEGs to each GO term in the GO database or each KEGG pathway in the KEGG database, and second calculated the number of genes in each GO term or KEGG pathway and finally applied a hypergeometric test to find a GO term or KEGG pathway that were significantly enriched in DEGs compared to the entire genome background. 

### 2.5. Quantitative Real-Time PCR (qRT-PCR)

The qRT-PCR was used to validate the accuracy of DEGs in L5D3 at the transcriptome level and the expression pattern of DEGs in the 5th instar larvae was investigated. Reverse transcription was performed by HiScript II QRT SuperMix (Vazyme, Nanjing, China) as follows: total RNA (1 μg) (without genome contamination) and 4 × gDNA Wiper were mixed with 5 × HiScript II QRT SuperMix and then incubated at 25 °C for 10 min, 50 °C for 15 min, and 85 °C for 2 min [14]. The cDNA was used for real-time PCR detection using the UltraSYBR Mixture (Cwbio, Beijing, China) and qRT-PCR was executed using the Light Cycler^®^ 480 System (Roche, Basel, Switzerland). The primer sequences for target genes were listed in Table S1 using primer 3.0 software (Premier Biosoft International, Palo Alto, CA, USA). The expression level of action A3, a silkworm housekeeping gene, was regarded as an internal reference in standardization [14]. Before Quantitative real-time PCR, the efficiency and specificity of primers were confirmed. The volume of the reaction system was 20 μL, containing 10 μL UltraSYBR Mixture, 1 μL cDNA, 0.5 μL forward and reverse primers and 8 μL ddH_2_O. The qRT-PCR program was as follows: initial denaturation at 95 °C for 10 min; followed by 40 times of denaturation at 95 °C for 10 s, annealing at 60 °C for 30 s, and extension at 72 °C for 32 s; a melting curve program was set at 95 °C for 15 s, 60 °C for 1 min, 95 °C for 15 s, and a60 °C for 15 s with a final cooling step at 4 °C for 30 s [23]. The data were analyzed with the LightCycler^®^96 software (Roche Diagnostics, Indianapolis, IN, USA) using the 2^−ΔΔCt^ method.

### 2.6. Statistical Analysis

The data were analyzed using one-way ANOVA data analysis. All the data were obtained in triplicate and presented as mean ± standard error (SE) (*n* = 3). Statistical significance at *p* < 0.05 was measured using SPSS 20.0 software (IBM, Endicott, NY, USA).

## 3. Results

### 3.1. Transcriptome Sequencing and Assembly

The deep sequencing of RNA from the fat body (5L3D) treated with H_2_S using the illumine sequencing and the data processing results were shown in Table 1. The raw data of H_2_S-treated and control groups were approximately 7.75 GB and 7.66 GB, respectively. After filtering and removing the low-quality reads, adaptors and poly-N stretches, the valid data and valid reads ratio of each replicate in H_2_S-treated group were obtained as follows: 7.55 Gb (99.12%), 7.87 Gb (98.91%), and 7.54 Gb (98.39%) and that of the control group were 7.42 Gb (94.00%), 7.37 Gb (98.99%), and 7.50 Gb (98.12%). The quality scores of Q30 exceeded 98% in each sample. Moreover, the mapped ratio in H_2_S-treated and control groups were 97.35%, 97.43%, and 97.72% and 93.19%, 97.35%, and 97.52%, respectively, which highly matched with the silkworm genome (Table S2). The expression interval of genes was mainly focused on the 0.3~3.57 FPKM interval (FI) and the ratio was about 35% gene of the control group and 39% of H_2_S-treated group (Table S3). These results revealed a good quality sequencing analysis and the genes were analyzed in the following section.

### 3.2. DEG Identification and Analysis

**Figure 1:**
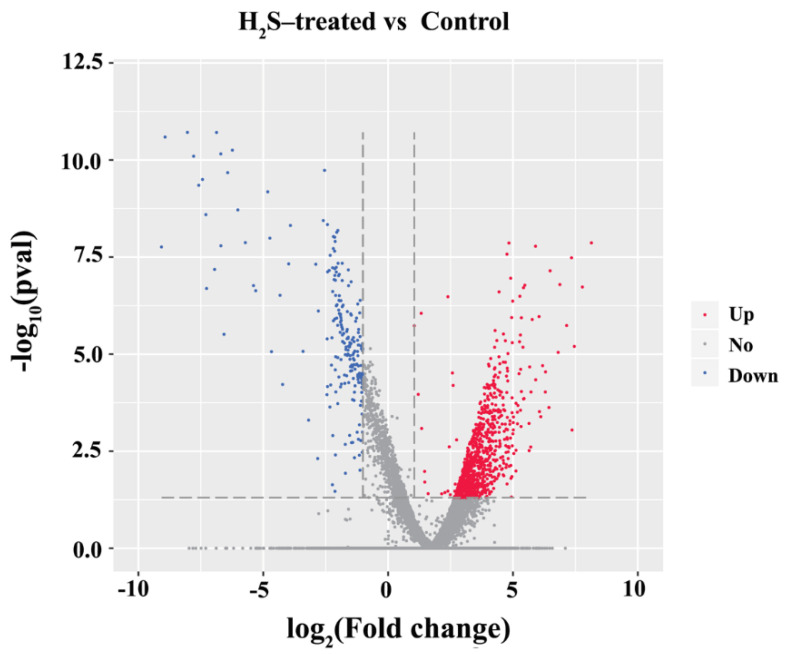
The volcano plot of DEGs. The red points represent the up-regulated genes, the blue points represent the down-regulated genes, and the grey points represent the genes without differential expression.

DEGs in H_2_S and control groups were calculated according to the FPKM value of the obtained genes and then a large number of genes were differentially expressed after H_2_S exposure. The volcano plot of DEGs was shown in Figure 1 and the detailed information of DEGs was listed in Table S4. A total of 1200 DEGs were obtained between the H_2_S-treated and control groups. The number of up-regulated and down-regulated DEGs was 977 and 223, and the up-regulated genes were higher than down-regulated genes. 

### 3.3. GO Analysis of DEGs

**Figure 2:**
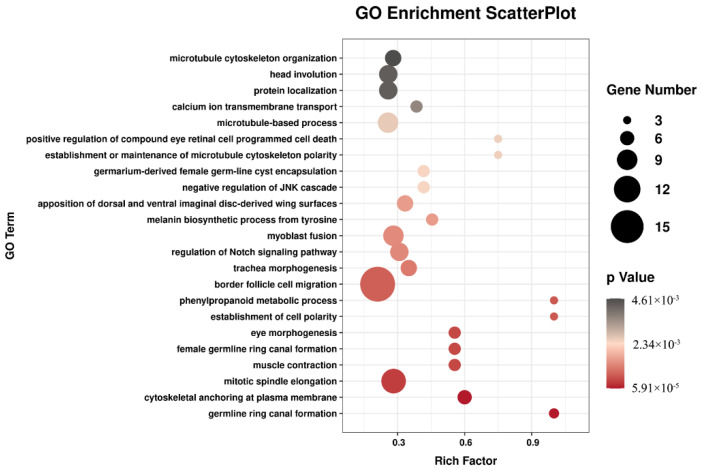
GO classification of DEGs (*p* ≤ 0.01).

The GO database can provide standard terms for describing the comprehensive properties of genes in organisms as an international classification system. In this study, DEGs were assigned to different GO categories such as biological processes, cellular components, and molecular functions. The major subcategories of DEGs for biological processes were border follicle cell migration, mitotic spindle elongation, myoblast fusion and microtubule-based processes, as well as others. (Figure 2 and Table S5). Through the results of the GO analysis, it was revealed that H_2_S could influence these multiple biological processes by regulating the expression of genes in the fat body of silkworms at the molecular level.

### 3.4. KEGG Pathway Analysis

**Figure 3:**
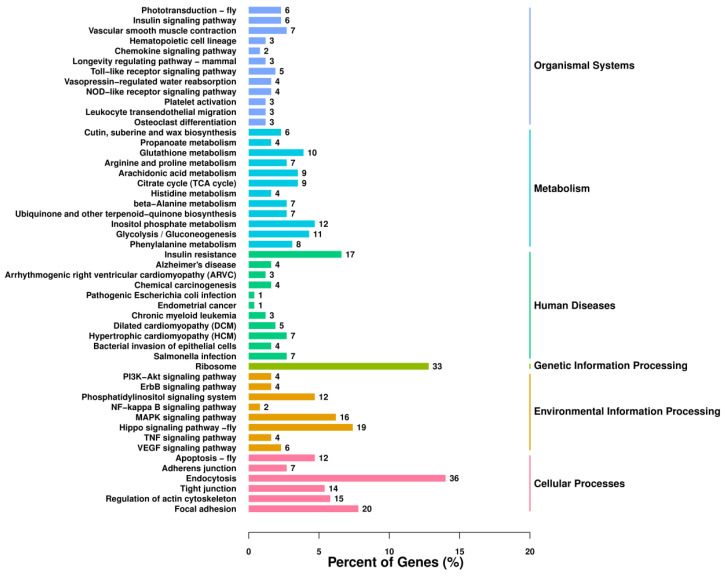
The KEGG classification of DEGs. The x-axis represents the number of genes annotated into the pathway and the proportion of the number of DEGs annotated to the total number of genes. The y-axis represents the name of the enriched KEGG pathways.

To systematically analyze the biological function and metabolic pathways of DEGs, KEGG pathway annotations were obtained. The 561 genes of 1200 DEGs predicted to encode enzymes were mapped into 225 KEGG pathways. These annotated genes were assigned to six categories including organismal systems (49 pathways), metabolism (74 pathways), human diseases (39 pathways), genetic information processing (19 pathways), environmental information processing (28 pathways), and cellular processes (16 pathways) (Figure 3). In the organismal systems, vascular smooth muscle contraction (7 DEGs) is the most abundant metabolic pathway followed by the insulin signaling pathway (6 DEGs) and toll-like receptor signaling pathway (5 DEGs). The most abundant metabolic category in metabolism is inositol phosphate metabolism (12 DEGs) followed by glycolysis/gluconeogenesis (11 DEGs), glutathione metabolism (10 DEGs), and the citrate cycle (9 DEGs). In human disease, it is mainly related to insulin resistance (17 DEGs). In the category of genetic information processing, it is mainly enriched in ribosomes (33 DEGs) closely related to synthetic genetic material. In environmental information processing, the most abundant pathways are the Hippo signaling pathway (19 DEGs), MAPK signaling pathway (16 DEGs), and phosphatidylinositol signaling system (12 DEGs). In cellular processes, 36 DEGs were mainly enriched in endocytosis followed by focal adhesion (20 DEGs) and regulation of the actin cytoskeleton (15 DEGs).

A significant enrichment analysis was further performed on the metabolic pathways of DEGs, and a total of 26 significantly enriched metabolic pathways were obtained (Table 2). The terms were enriched to multiple molecular pathways such as the transport pathway and cellular community, including endocytosis, focal adhesion, regulation of the actin cytoskeleton, tight junction, and adherens junction; amino acid and carbohydrate metabolisms, such as glycolysis/gluconeogenesis, the citrate cycle (TCA cycle), phenylalanine metabolism, and histidine metabolism; immune-associated signal transduction such VEGF signaling pathway, the TNF signaling pathway, NOD-like receptor signaling, the MAPK signaling pathway, the Hippo signaling pathway-fly, the Toll-like receptor signaling pathway, and the NF-κB signaling pathway (Table S6). The top 10 metabolic pathways affected by H_2_S were endocytosis, ribosome, focal adhesion, the Hippo signaling pathway, the MAPK signaling pathway, regulation of the actin cytoskeleton, tight junction, inositol phosphate metabolism, glycolysis/gluconeogenesis, and the TCA cycle (Table 2). These annotations indicated that H_2_S could influence the multiple biological pathways in the fat body of silkworms and provide a new perspective to the effects of H_2_S on silkworms.

### 3.5. The Validation of DEGs by qRT-PCR

**Figure 4:**
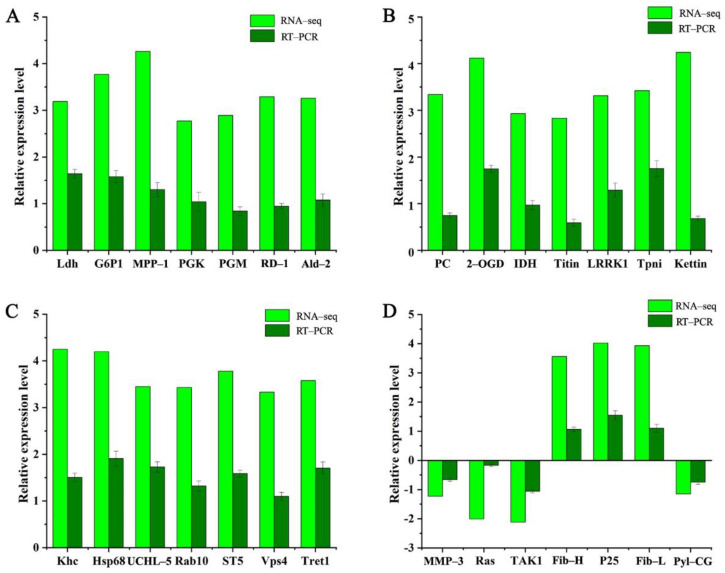
The qRT-PCR validation for DEGs expression after exposure to H_2_S. DGEs related to the glycolysis pathway (**A**), the TCA cycle (**B**), endocytosis (**C**), and inflammation and silk protein synthesis (**D**) were analyzed. The full names of abbreviations of genes were listed in Table S1.

To validate the accuracy of DEGs obtained from the transcriptome between the H_2_S-treated and control groups, 28 candidate genes were selected for qRT-PCR analysis. The selected DEGs were mainly annotated to glycolysis/gluconeogenesis, the TCA cycle, focal adhesion, endocytosis, adherens junction, the NF-κB signaling pathway, the MAPK signaling pathway, and the synthesis of fibroin. Good consistency between the results of qRT-PCR and the transcriptome confirmed the accuracy and reliability of the sequencing data and revealed the significant difference of these genes during H_2_S exposure (Figure 4 and Table S3). 

### 3.6. The Expression Pattern Analysis of DEGs

**Figure 5:**
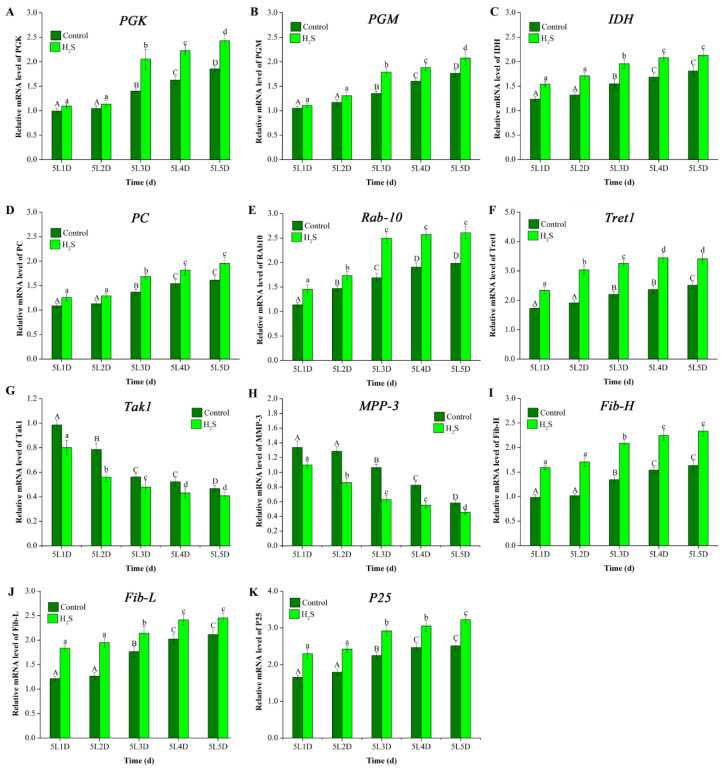
Expression changes of 10 DEGs after H_2_S exposure at different time points in the 5th instar larvae (5L1D, 5L2D, 5L3D, 5L4D, and 5L5D). (**A**) PGK; (**B**) PGM; (**C**) IDH; (**D**) PC; (**E**) Rab-10; (**F**) Tret1; (**G**) Tak1; (**H**) MPP-3; (**I**) Fib-H; (**J**) Fib-L; and (**K**) P25. The lowercase letters (a, b, c, and d) represent the significant difference at *p* ≤ 0.05 among the expression levels at different times of the H_2_S treated group; the uppercase letters (A, B, C, and D) represent the significant difference at *p* ≤ 0.05 among the expression levels at different times of the control group. The full names of abbreviations of genes are listed in Table S1.

A total of 11 DEGs was representatively selected to analyze the expression level pattern in the 5th instar larvae using qRT-PCR. Genes (relating to the glycolytic pathway and TCA cycle) such as lactate phosphoglycerate kinase (PGK), phosphoglucomutase (PGM), NADP-dependent isocitrate dehydrogenase (IDH), and pyruvate carboxylase (PC) in the H_2_S-treated group were up-regulated compared to control group (Figure 4A,B). Meanwhile, the levels of these genes were significantly increased at the 5L3D, 5L4D, and 5L5D comparing with 5L1D in the H_2_S group (Figure 5A–D). In addition, the expression levels of the members of the Rab family of small GTPases (Rab10) and trehalose transporter 1 (Tret1) in endocytosis were up-regulated after H_2_S treatment (Figure 4C). The expression levels of Rab10 and Tret1 were significantly increased in a time-dependent manner (Figure 5E,F). On the contrary, transforming growth factor-β activated kinase1 (Tak1) and matrix metalloproteinase-3 (MMP-3) were significantly decreased in the 5th instar larvae after H_2_S exposure (Figure 5G,H). Moreover, the increased expression of fibroin synthesis genes, such as the fibroin heavy chain (Fib-H), fibroin light chain (Fib-L), and glycoprotein P25 (P25) was observed during the 5th larvae stage after H_2_S exposure (Figure 4D). At the end of the 5th instar larvae, the expression levels were higher than the early 5th instar larvae (Figure 5I–K).

## 4. Discussion

H_2_S has been accepted as the third transmission gas followed by nitric oxide (NO) and carbon monoxide (CO) in recent years with applications to various insects, such as *C. elegans* [7,8,9,10], *D. melanogaster* [11,12], and *B. mori* [13]. RNA-seq technology is widely used in the field of silkworms to investigate the physiological and biochemical changes at the transcriptional level under diverse conditions [4,14,24]. In our previous research, it was found that the development (body weight) and economic characteristics (cocoon weight, cocoon shell ratio, and cocoon shell weight) were increased after continuous treatment with 7.5 μM of H_2_S. However, there is no effective report for the effects of H_2_S on the silkworm at the transcriptional level [13]. In the present study, the expression levels of genes in the fat body of silkworms after exposure to H_2_S was investigated using transcriptomic and bioinformatic analysis. The results showed significantly different responses to H_2_S and provided useful insights into the role of H_2_S on silkworms. 

Using transcriptome, a total of 1200 DEGs were identified in the fat body of silkworms at the 5th instar larvae. After treatment with H_2_S, up-regulated DEGs (977) were higher than the down-regulated DEGs (223). These DEGs were mainly involved in glycolysis/gluconeogenesis, endocytosis, and the TCA cycle. The carbohydrate metabolism is one of the important energy metabolism pathways in organisms, incorporating glycolysis/gluconeogenesis, the TCA cycle, oxidative phosphorylation, and the pentose phosphate pathway [25]. Energy metabolism, especially the metabolic control of carbohydrates, is essential for insect growth and development [26]. Glucose is oxidized to pyruvate in glycolysis/gluconeogenesis [25]. Glycolytic activity is regulated by many enzymes in the glycolytic pathway such as PGK and PGM. PGM can convert glucose 1 phosphate into glucose 6 phosphate, providing the raw materials for the glycolytic pathway [27]. PGK is commonly known as a soluble and membrane-bound protein in different organisms [27]. In one of the glycolytic processes, phosphorylation of 1,3-diphosphoglycerate to 3-phosphoglycerate is catalyzed by PGK [28]. Like glycolysis/gluconeogenesis, the TCA cycle also provides the energy and intermediates for diverse biosynthetic pathways [25]. IDH, one of three rate-limiting enzymes in the TCA cycle, catalyzes the TCA cycle reaction wherein isocitrate is oxidized and decarboxylated to produce α-ketoglutarate, NADH, and CO_2_ [29]. PC catalyzes the carboxylation of pyruvate into oxaloacetate for gluconeogenesis, the urea cycle, lipid synthesis, and other pathways which are the primary anaplerotic pathways for the TCA cycle where the intermediates are replenished by alanine and lactate [30]. In this study, the results showed that the levels of PGM, PGK, IDH, and PC were up-regulated after H_2_S exposure. According to the expression pattern analysis of these genes, gene levels had gradually increased in a time-dependent manner. Especially for 5L4D and 5L5D, a significant difference was observed compared to 5L1D. These results indicated that the expression levels of the genes involved in carbohydrate metabolism were increased with H_2_S exposure, suggesting H_2_S can accelerate the carbohydrate metabolism of silkworms and provide much energy for silkworm metamorphosis.

The uptake and sorting of eukaryotes and the following recycling or degradation of fluid, membranes, membrane proteins, and macromolecules are regulated by endocytosis [31]. Endocytosis often depends on clathrin and occurs at clathrin-coated pits [32]. Utilizing endocytosis with clathrin-dependent or clathrin-independent uptake mechanisms, cargo proteins and adaptor molecules are delivered to early endosomes and sorted [33]. The Rab family of small GTPases can mediate membrane the trafficking processes of intracellular compartmentation, generation, and maintenance [34]. Rab10, which is closely related to clathrin-independent endocytosis is largely expressed in many model animals directing the trafficking of proteins. In *Caenorhabditis elegans*, Rab10 was required in interneuron postsynaptic glutamate receptor recycling and the transport between early endosomes and recycling endosomes [35]. Rab10 in the H_2_S-treated group was up-regulated compared to the control group, which indicated that the H_2_S-treated silkworms were more active in supporting endocytosis for the body. Tret1 participated in the transfer of trehalose, the major hemolymph sugar in most of the insect, which is synthesized in the fat body and released into the hemolymph. The transportation of trehalose with the up-regulated expression level of Tret1 after exposure to TiO_2_ nanoparticles was accelerated in the silkworm, showing that TiO_2_ nanoparticles could facilitate carbohydrate and the nutrition metabolism [36]. In this study, the level of Tret1 was up-regulated with the H_2_S supplement to accelerate the transport rate of trehalose. Moreover, the expression levels of Rab10 and Tret1 were increased in a time-dependent manner during the 5th larvae. These results showed that H_2_S could accelerate the transmission of mass in the silkworm fat body via up-regulating the expressions of Rab10 and Tret1 to provide material for larva–pupae metamorphosis.

Previous studies reported that H_2_S exerted anti-inflammatory effects on T lymphocytes. Rats injected with NaHS inhibited inflammatory processes such as leukocyte infiltration and adherence to the vascular endothelium [37]. In this study, DEGs were annotated to various pathways related to inflammation by KEGG analysis, including the TNF signaling pathway, the MAPK signaling pathway, and the NF-κB signaling pathway. 

Tak1 is a serine/threonine kinase and a member of the mitogen-activated protein kinase kinase (MAP3K) family which is activated by receptors such as TGF-β, TNF-α, and IL-1. MMP-3 is a matrix-soluble protein containing the binding site of NF-κB and can participate in the degradation of ECM and activate the precursors of MMPs. The content of MMP-3 was increased in the mouse arthritis model and the administration of baicalin can reduce the gene expression level of MMPs and alleviate inflammatory processes [38]. After H_2_S exposure, levels of Tak1 and MMP-3 were down-regulated compared to the control group. Moreover, the expression levels of Tak1 and MMP-3 at the end of 5th instar larvae were significantly decreased compared to the early 5th instar larvae. These results indicated that H_2_S might have an anti-inflammatory role in silkworm growth.

In addition, significant changes in the genes related to fibroin synthesis were observed in this study. The domestic silkworm has tremendous economic value and the most important manifestation of its value is silk production [39]. The function of the silk gland is to synthesize silk protein and during the 5th instar larvae it is the biggest organ of *B. mori* [39]. It was reported that Fib-H, Fib-L, and P25 encoding fibroin were expressed in all the larval instars. However, their highest expression was noticed before the last molting period [40]. The expression levels of Fib-L, P25, and Fib-H were up-regulated in response to H_2_S compared to the control group. In addition, the expression of Fib-H at the end of the 5th instar larvae (5L4D, 5L5D) was rapidly increased and higher than on the other days. Similarly, the expression levels of P25 and Fib-L were also significantly increased. These results indicated that H_2_S could promote the fibroin synthesis by up-regulating the expression levels of Fib-L, P25, and Fib-H, which was beneficial to silk production. These results were similar to the previous study which reported the up-regulated expression of Fib-L, P25, and Fib-H after treatment with titanium dioxide nanoparticles (TiO_2_NPs) [41].

## 5. Conclusions

To summarize, this is the first-ever report to investigate the fat body of silkworms at the transcriptional level after H_2_S exposure using the transcriptome analysis during the 5th larvae stage. Transcriptome analysis could detect numerous genes and signaling pathways and most of the DEGs were up-regulated compared to the control group. The expression of key genes related to energy metabolism, transport pathways, and fibroin synthesis in silkworms after H_2_S exposure was up-regulated; conversely, the genes involved in inflammatory processes were down-regulated, which indicated that H_2_S may lead to the promotion of biological processes such as energy and nutrition metabolism and inhibition of inflammatory processes. These results provide a novel approach to comprehending the molecular mechanisms underlying the H_2_S effects in the silkworm fat body. 

## Figures and Tables

**Table 1:** The sequencing data of samples.

Samples	FB_Control_1	FB_Control_2	FB_Control_3	FB_H_2_S_1	FB_H_2_S_2	FB_H_2_S_3
Raw reads	52,598,692	49,647,104	50,987,488	50,804,424	53,068,042	51,108,946
	(7.89 Gb)	(7.45 Gb)	(7.65 Gb)	(7.62 Gb)	(7.96 Gb)	(7.67 Gb)
High quality reads	49,443,624	49,144,228	50,029,926	50,357,636	52,490,302	50,286,178
	(7.42 Gb)	(7.37 Gb)	(7.50 Gb)	(7.55 Gb)	(7.87 Gb)	(7.54 Gb)
High quality reads ratio (%)	94.00	98.99	98.12	99.12	98.91	98.39
Q30 (%)	98.53	99.08	99.07	98.93	98.98	99.12
GC content (%)	50	48	49	48	48.50	48
Total mapped	46,077,049	47,843,354	48,788,614	49,023,730	51,140,817	49,141,008

**Table 2:** The main enriched KEGG pathways (*p* < 0.05) in the H_2_S-treated and the control groups.

Pathway ID	Pathway Name	Number of DEGs	*p*-Value
ko03010	Ribosome	33	<0.01
ko04510	Focal adhesion	20	<0.01
ko05132	Salmonella infection	7	<0.01
ko04370	VEGF signaling pathway	6	<0.01
ko04810	Regulation of actin cytoskeleton	15	<0.01
ko00360	Phenylalanine metabolism	7	<0.01
ko04530	Tight junction	13	<0.01
ko04668	TNF signaling pathway	4	0.01
ko00010	Glycolysis/Gluconeogenesis	11	0.01
ko04144	Endocytosis	35	0.01
ko00562	Inositol phosphate metabolism	12	0.01
ko00130	Ubiquinone and other terpenoid-quinone biosynthesis	6	0.01
ko04380	Osteoclast differentiation	3	0.01
ko04670	Leukocyte transendothelial migration	3	0.01
ko00410	beta-Alanine metabolism	7	0.01
ko05100	Bacterial invasion of epithelial cells	4	0.02
ko04611	Platelet activation	3	0.02
ko04520	Adherens junction	7	0.02
ko04621	NOD-like receptor signaling pathway	4	0.02
ko04391	Hippo signaling pathway-fly	19	0.02
ko04010	MAPK signaling pathway	16	0.03
ko00340	Histidine metabolism	4	0.04
ko05410	Hypertrophic cardiomyopathy (HCM)	6	0.04
ko04064	NF-kappa B signaling pathway	2	0.04
ko00020	Citrate cycle (TCA cycle)	9	0.04
ko04962	Vasopressin-regulated water reabsorption	4	0.04

## Data Availability

The raw data in present paper was deposited in NCBI with the submission ID SUB10721255 (http://www.ncbi.nlm.nih.gov/bioproject/783962, 27 November 2021).

## Supplementary Materials

The following are available online at https://www.mdpi.com/article/10.3390/insects12121110/s1, Tables S1–S6. Table S1. Primer’s sequence of genes used in qRT-PCR analysis. Table S2. Statistical analysis of the RNAs Libraries. Table S3. Distribution of gene expression levels between H_2_S-treated and the control group and the partial DEGs. Table S4. The list of DEGs comparison between H_2_S treated group and control group. Table S5. Analysis of the enriched GO terms. Table S6. Analysis of the enriched KEGG pathway.
