# Supplementary material for: Transcriptome Analysis Reveals the Gene Expression Changes in the Silkworm (Bombyx mori) in Response to Hydrogen Sulfide Exposure
Source: Insects. 2021 Dec 13;12(12):1110. doi: 10.3390/insects12121110 (PMC8706860; doi:10.3390/insects12121110)
Supplement: Supplementary file 1 [file insects-12-01110-s001.zip › Table S1.pdf]

Table S1. Primer's sequence of genes used in qRT-PCR analysis.

Gene ID	Gene Name	Primers
100145915	Actin A3	F: aagattccgttgcccagagg<br>R: atgtccacgtcgcacttcat
TpnI	Troponin I transcript variant C	F: ggagccaggggaaacgtaaa<br>R: ttgggagttcaggtcggaga
LOC105842021	Ras GTPase- activating protein nGAP-like	F: ctaaggtatccggcagcagg<br>R: acttcccgttcggttctgac
100529234	Transform growth factor beta-activated kinase 1 (Tab1)	F: ctgatcctagtaagcgcggg<br>R: acggccttgaagtctgtctg
Ldh	Lactate dehydrogenase	F: cgttcacgcggtctctactt<br>R: gatttccgaagctgtgccac
LOC101738228	Multiple inositol polyphosphate phosphatase 1-like (Mpp-1)	F: cgtgaccgtgtttctaagcg<br>R: atcccctctggctgcattat
LOC101739645	Phosphoglycerate kinase (PGK)	F: tgctggcgtgtttgagtttg<br>R: agtagagacgtgcgagacct
LOC101740694	Retinal dehydrogenase 1-like (RD-1)	F: ctactggcaggtggagaacg<br>R: gctctggactggaccgaaaa
LOC101743212	Phosphoglucomutase (PGM)	F: ttccgatgctaccaccgatg<br>R: ttcgactgtaaactgccgct
LOC692988	Aldehyde dehydrogenase isoform 2 (Ald-2)	F: tcgtaataggcgcctggaac<br>R: aactcagtggtctcttgcgg
LOC101736196	Glucose-6-phosphate 1-epimerase (G6P1)	F: gcggaagatgcggatacaga<br>R: atgttggggaactcgtcgtc
Khc	Kinesin heavy chain	F: gcgttctggtgagactgtca<br>R: caggaggtgctggaggtttt
LOC100862772	Heat shock protein 68 (Hsp68)	F: cattgaaactgccggtggtg<br>R: tgctggttggctgtcagaat
LOC101735878	Ubiquitin carboxyl-terminal hydrolase 5 (UCJHL-5)	F: tcgccataggagttgaggga<br>R: accattgtccagctgcttga
Rab10	Small GTP-binding protein Rab10 (Rab 10)	F: acgagcatgcaaacgaggat<br>R: tccgctagttccgagaaagc
ST5	Putative sugar transporter protein 5	F: ggtgtacacgtgcgaggtat<br>R: cagcatgctcaggagtgaca
Vps4	Vacuolar protein sorting 4	F: agcggctaaaagtgagggat<br>R: tgacattgtaactggcggct
LOC101735597	Protein yippee-like CG15309 isoform X1 (Pyl-CG15309)	F: agacaatgctcggctggaaa<br>R: ccaaccgttctccttcacca
LOC101740132	Leucine-rich repeat serine/threonine-protein kinase 1 (LRRK1)	F: gagtctaatgcctcgtcccg<br>R: ttcctccacgtctagcagga
LOC101745176	Titin	F: cagtggaaagtttggcgtgg<br>R: aattcggcgtctctgttcgt
LOC101744194	Facilitated trehalose transporter Tret1 (Tret1)	F: gtatccactcggcagctacc<br>R: aatagacctgtgacggagcg
Kettin	Kettin protein	F: gacgaggatagagccggttg<br>R: agtagccctgggagttacgt
LOC101736589	Matrix metalloproteinase-3-like (MMP-3)	F: ccggagttggctacacttacac<br>R: cctttctatgcgatccttgct
LOC100101164	NADPH-specific isocitrate dehydrogenase (IDH)	F: atgtggctaagtccgaacgg<br>R: cgcctctctgtcgtaccatc
LOC101736848	2-Oxoglutarate dehydrogenase, mitochondrial (2-OGD)	F: ggagacaacgagggcaagaa<br>R: tcacgtggaatatcggagcg
LOC101737640	Pyruvate carboxylase (PC)	F: gggaggagctacattcgacg<br>R: atgcccagtaccaagttcgg
Fib-H	Fibroin heavy chain	F: gttacggagctggcaggg<br>R: agcaattcacacaaggcagt

P25	Fibroin P25	F: cgaaatgtatcccgggcaga<br>R: tcaaagttcagccacggaca
Fib-L	Fibroin light chain	F: gccggcttcagacaatctct<br>R: tgagcggttatgtaggcagc
