# Supplementary material for: Transcriptome Analysis Reveals the Gene Expression Changes in the Silkworm (Bombyx mori) in Response to Hydrogen Sulfide Exposure
Source: Insects. 2021 Dec 13;12(12):1110. doi: 10.3390/insects12121110 (PMC8706860; doi:10.3390/insects12121110)
Supplement: Supplementary file 1 [file insects-12-01110-s001.zip › Table S3.pdf]

Table S3. Distribution of gene expression levels between H_2S-treated and the control group.

FPKM Interval (FI)	0-0.1 FI	0.1-0.3 FI	0.3-3.57 FI	3.57-15 FI	15-60 FI	>60 FI
FB_Control_1	2843(29.20%)	2039(20.94%)	3224(33.11%)	842(8.65%)	365(3.75%)	423(4.34%)
FB_Control_2	2685(27.51%)	1926(19.73%)	3522(36.09%)	816(8.36%)	406(4.16%)	405(4.15%)
FB_Control_3	2860(27.91%)	1982(19.34%)	3576(34.89%)	967(9.44%)	431(4.21%)	433(4.22%)
FB_ H_2S _1	1953(16.47%)	1704(14.37%)	4591(38.72%)	2152(18.15%)	816(6.88%)	642(5.41%)
FB_ H_2S _2	2264(20.27%)	1844(16.51%)	4283(38.35%)	1567(14.03%)	641(5.74%)	569(5.09%)
FB_ H_2S _3	2004(17.75%)	1806(16.00%)	4573(40.51%)	1693(15.00%)	674(5.97%)	539(4.77%)

RNA-seq of the partial DEGs

Gene	$log_2(FC)$	*P*-Value	Regulation	Gene	$log_2(FC)$	*P*-Value	Regulation
MMP-3	-6.70	0.00	down	VPS4	3.33	0.01	up
Ras GAPs	-7.27	0.00	down	Pyl-CG15309	-1.15	0.00	down
Ldh	3.19	0.02	up	Titin	2.83	0.05	up
MPP-1	3.25	0.02	up	LRRK1	3.31	0.01	up
Tab1	-6.87	0.00	down	Tret1	4.02	0.00	up
G6P1	3.77	0.02	up	Kettin	4.24	0.00	up
PGK	2.77	0.04	up	2-OGD	3.42	0.01	up
PGM	2.89	0.02	up	Fib-H	3.56	0.02	up
Ald-2	3.26	0.00	up	P25	4.02	0.00	up
Khc	4.25	0.00	up	PC	2.97	0.02	up
Hsp68	4.20	0.00	up	TpnI	3.42	0.02	up
UCHL-5	3.45	0.00	up	IDH	2.93	0.03	up
Rab10	4.08	0.01	up	Fib-L	3.93	0.03	up
ST5	3.78	0.02	up	RD-1	2.77	0.04	up
